# Serum and cerebrospinal fluid biomarker profiles in acute SARS-CoV-2-associated Guillain–Barré syndrome

**DOI:** 10.1093/braincomms/fcab219

**Published:** 2021-09-25

**Authors:** José Berciano

**Affiliations:** 1 University of Cantabria, Santander, Spain; 2 University Hospital “Marqués de Valdecilla (IDIVAL)”, Santander, Spain; 3 “Centro de Investigación Biomédica en Red de Enfermedades Neurodegenerativas (CIBERNED)”, Santander, Spain

I read with great interest the paper by Paterson and colleagues^1^ evaluating serum and CSF biomarkers in acute SARS-COVID-2-associated neurologic disorders including Guillain–Barré syndrome (GBS). Concerning GBS, they found that levels of neurofilament light protein were elevated in serum, but normal in CSF. Wisely, the authors state that such finding implies that neurofilament light protein is released from peripheral nerves into blood and does not freely pass back into CSF. I wish to offer the pathological basis for this proposal.

In very early GBS (≤4 days after onset), the main pathogenic lesion is endoneurial inflammatory oedema predominating in nerve segments with less efficient blood–nerve barrier, including spinal nerve roots, spinal ganglia, spinal nerves, and pre-terminal and terminal nerve segments.^2^ In nerve segments possessing epi-perineurium, namely external to subarachnoid angle and particularly in spinal nerves, oedema might be pathogenic by means of increasing endoneurial fluid pressure, which constricts transperineurial microcirculation producing rapid ischaemic nerve injury, potential cause of distally accentuated Wallerian-like degeneration. Figure  1 illustrates drastic change of pathology between ventral spinal root and ventral ramus of spinal nerve at lumbar level.^3^ Ultrasonographic studies have revealed that oedema of ventral rami of spinal nerves is a hotspot in early GBS, either demyelinating or axonal.^4^ In severe GBS, massive axonal damage in ventral rami of spinal nerves may be followed by retrograde axonal degeneration extending into ventral roots, eventually causing central chromatolysis on the anterior horn cells.^2^ Needless to say, in acute motor axonal neuropathy, Wallerian-like degeneration may also be accounted for by anti-ganglioside antibodies.

**Figure 1:**
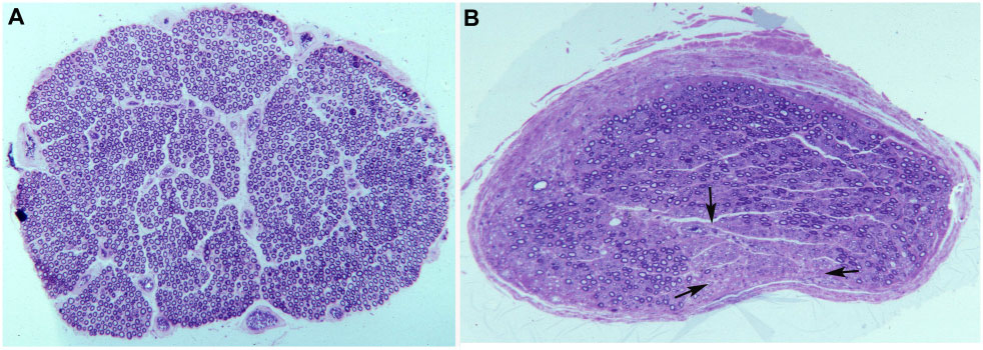
Pathological findings in a fulminant GBS patient who died on Day 60. Electrophysiological studies (Days 4, 17 and 50) initially showed normal nerve conduction velocities with further slowing, progressive attenuation of compound muscle action potentials and profuse denervation. **A**, The density of myelinated fibres appears preserved in this complete, semithin cross-section of L5 ventral root; note the absence of perineurium (×62 before reduction). **B**, In this complete cross-section of ventral ramus of L3 nerve, there is widespread diminution of myelinated fibres, particularly in sub-perineurial regions. Note the presence of a wedge-shaped area exhibiting almost complete loss of myelinated fibres (arrows), a finding characteristic of endoneurial ischaemia (×62 before reduction). Adapted from Berciano and colleagues.^3^

Assuming that the aforementioned increase of serum neurofilament light protein was detected in the early stages of GBS/COVID-19, I would suggest that it is correlated with axonal dysfunction caused by inflammatory oedema in extradural nerve trunks. Conceivably, in the course of the disease extension of axonal changes to spinal nerve roots could account for potential neurofilament light protein changes in CSF.

## Data availability

Data sharing is not applicable to this article as no new data were created or analysed.

## Competing interests 

The author reports no competing interests.
